# Attitudes towards AI counseling: the existence of perceptual fear in affecting perceived chatbot support quality

**DOI:** 10.3389/fpsyg.2025.1538387

**Published:** 2025-08-01

**Authors:** Wing Man Keung, Tsz Yan So

**Affiliations:** Department of Psychology, The University of Hong Kong, Pokfulam, Hong Kong SAR, China

**Keywords:** attitudes towards AI counseling, AI anxiety, chatbot counseling, emotional support, stress, confirmation bias, perceived support quality

## Abstract

**Introduction:**

Due to the shortage of financial and human resources in the local mental health industry, AI counseling presents itself as a cost-effective solution to address this limitation. However, fear and concerns about AI may hinder the adoption of AI in counseling. This study examined the relationships between individuals’ prior AI exposures, AI anxiety levels, attitudes towards AI, and their perceived support satisfaction with the counseling chatbot.

**Methods:**

With a simulated counseling chatbot developed using Azure OpenAI GPT-4 model (1106-preview version) and a sample of 110 local Chinese in Hong Kong, this study explored the potential existence of perceptual fear in affecting people’s perceived support quality of the chatbot by manipulating the informed perceptual labels—Told-Human (told to be receiving human counseling) and Told-AI (told to be receiving AI counseling).

**Results:**

Perceptual fear of AI adversely affected participants’ perceived support quality of the counseling chatbot, *t* (108) = 2.64, *p* = 0.009, BCa 95% CI = [0.186, 1.342], with Hedges’ correction of 1.55. While the significant reduction in stress levels demonstrated the chatbot’s implicit capability in providing emotional support (*p* = 0.03), participants showed explicit reservations about its helpfulness.

**Discussion:**

This study highlights the importance of accounting for the influence of individuals’ pre-existing beliefs on the perceived support quality of counseling chatbots. Future cross-cultural studies with a larger sample may shed more light by investigating dynamic intervention approaches and conducting sentiment and thematic analyses of client-chatbot conversations.

## 1 Introduction

The ability of Artificial Intelligence (AI) to learn from vast datasets and enhance its performance over time makes it increasingly capable of mimicking a wide range of human characteristics and quickly becoming effective in jobs that used to be a human prerogative (Kaplan and Haenlein, 2019; Prabu et al., 2014). The consideration of incorporating AI into counseling arises due to the shortage of financial and human resources to handle the high demand for services worldwide, where people seeking psychological help often face long waiting times (Stringer, 2023). In Hong Kong, the waiting times for psychiatric services can exceed 100 weeks in some areas (Hospital Authority, 2024). Without timely support, their issues may be exacerbated, necessitating more intensive intervention and placing an even higher burden on an already overwhelmed system. It calls for a relieving countermeasure to deal with this vicious cycle in the mental health system.

AI can be used simultaneously by multiple users and thus does not require massive financial and human resources to reach the service demand. It is also suggested that AI-based psychotherapy could enable clients to share embarrassing events and confess emotions more comfortably as it does not involve face-to-face meetings (Aktan et al., 2022; Lucas et al., 2014). It can provide 24/7 support without geographical barriers (Shankar Ganesh and Venkateswaramurthy, 2025), while responses generated by GPT-4 were found competitive with those of human counselors (Inaba et al., 2024). Recent advancements in chatbots based on large language models and GPT have also significantly impacted psychological intervention and counseling by enhancing personalization and efficacy. These include the rapid advancements in GPT-4, which allow increasingly sophisticated and more empathetic responses (Moell, 2024), the incorporation of multimodal interactions, such as voice interaction in Amanda (Vowels et al., 2025), and real-time AI-driven mood detection available in Gemini (Syarifa et al., 2024). While most mental health chatbots have been designed for depression and anxiety, an increasing number of specialized chatbots are being developed, for example, for diagnosing autism (Mujeeb et al., 2017) and supporting clients with substance abuse (Prochaska et al., 2021). Despite these benefits and the capability of AI, its adoption in the mental health system raises concerns. These concerns include the potential displacement of human healthcare professionals (Espejo et al., 2023), algorithmic biases in AI psychotherapy (Brown and Halpern, 2021; Denecke et al., 2021; Knox et al., 2023), cybersecurity issues (Aktan et al., 2022; Lee et al., 2021), and the lack of human emotions and reciprocal affect (Brown and Halpern, 2021; Fiske et al., 2019). The dichotomy motivates ongoing investigations into public attitudes towards AI counseling.

### 1.1 Prior exposure to dual-perspective information

The development of AI has been the subject of considerable debate, with various perspectives and attitudes emerging regarding its advancement. These attitudes may be developed from prior AI exposures. Inspired by the advancement of AI, science fiction movies have been featuring it as a central narrative element since the mid-20th century. While some portray AI in a positive light (e.g., *Alita: Battle Angel*, *Big Hero 6*, *The Iron Giant*), others depict AI as antagonistic, dangerous and manipulative, which has become conscious and desires to surpass humans (e.g., *The Terminator*, *Transcendence*, *Ex Machina*). Likewise, some news reports suggested that AI is undesirable—for example, the prediction that jobs would be eliminated and inequality would worsen due to the occurrence of AI (Cerullo, 2024; Milmo, 2024), high-profile data breaches (Boutary, 2023; Hsu, 2024), and biased responses from AI leading to discrimination issues (Lytton, 2024; Milmo and Hern, 2024)—while some reported the benefits of AI such as the extraordinary role of AI in healthcare settings (Stekelenburg, 2024) and the potential reduction of taxes when AI is adopted (Parton, 2024). While the media plays a significant role in influencing people’s attitudes (Hanewinkel et al., 2012; Perciful and Meyer, 2017; Wang et al., 2021), dual-perspective information introduces uncertainty and ambivalent attitudes towards AI.

Meanwhile, research has shown that negative information typically receives more attention and exerts a stronger influence than positive information (Robertson et al., 2023; Zollo et al., 2015). This is due to the negativity bias in human psychology that occurs cross-culturally (Soroka et al., 2019). Unpleasant exposures can have a more profound impact on one’s psychological state and evaluations than pleasant ones, even when the magnitude of their emotions is equal (Rozin and Royzman, 2001). The inherent attraction to negativity motivates an examination of how individuals’ exposure to AI shapes their attitudes towards AI.

### 1.2 How unpleasant exposure shapes attitudes and triggers AI anxiety

High frequency, unpleasant emotional valence and strong immersion during AI exposures may reinforce this impact and help explain individuals’ unfavorable attitudes towards AI. It is well established that media exposure can influence people’s perceptions and evaluative processes (Kirkpatrick et al., 2024; Nguyen et al., 2024). Public awareness of AI-related threats and risks can be fostered through the widespread sharing of information online (Kirkpatrick et al., 2024), which may lead to the development of negative attitudes toward AI and hinder its adoption (Hasan et al., 2021; Vu and Lim, 2021). It is also found that more attention paid to AI content is associated with higher levels of economic risk perceptions regarding AI, including job replacement and dependency on AI (Kirkpatrick et al., 2023). These findings underscore the media’s role in shaping public perceptions and attitudes toward AI.

From a Pavlovian behavioral perspective, when people encounter more negative information about AI, the neutral stimulus “AI” becomes associated with negative consequences (i.e., unconditioned stimuli such as job loss and cybersecurity issues) that trigger unpleasant feelings like fear and anger (i.e., unconditioned responses). The successful association between the neutral stimulus and undesirable consequences and feelings could be explained by the activation of similar neural pathways as those activated by the unconditioned stimuli (Gore et al., 2015; Lanuza et al., 2008). As a result, people form attitudes towards AI that align with the affective value associated with it. From Darwin’s evolutionary perspective (Ekman, 2009), the conditioned fear of AI serves as an adaptive and evaluative signal that triggers fight-or-flight reactions in response to AI threats. Fight-or-flight reactions resemble people’s general attitudes towards AI nowadays—some people would fight, confronting and manipulating AI to address its flaws without letting it become a threat to humans, while others may choose to flee, discouraging the incorporation of AI into society. It reflects that the unpleasant emotions aroused by AI exposure can inevitably have adverse effects on individuals’ acceptance of AI, due to the survival instincts we inherit. Along with emotional engagement, a stronger immersion in the exposure can make information more persuasive and memorable, which enhances message internalization that facilitates attitude change (Green and Brock, 2000; Valkenburg and Peter, 2013).

Higher frequency, more unpleasant emotions, and stronger immersion in AI exposure could also contribute to the development of negative schemas surrounding AI. Individuals’ excessive fear or concerns about AI in their personal or social lives are referred to as AI anxiety (Wang and Wang, 2022). It includes four aspects—(1) *Job replacement anxiety* refers to the fear of AI replacing their jobs; (2) *Sociotechnical blindness* refers to the anxiety of technological determinism and the lack of understanding that AI depends on humans; (3) *AI Configuration anxiety* denotes the fear of humanoid AI, and (4) *AI learning anxiety* denotes individuals’ fear of learning AI technologies (Wang and Wang, 2022). The existence of AI anxieties implies that information from prior unpleasant AI exposure introduces personally relevant threats to self-interest and well-being, and becomes integrated into our cognitive schemas, forming anxieties related to AI.

Existing studies have investigated the relationships between AI awareness and AI anxiety in organizational contexts (Elfar, 2025; Kong et al., 2021; Zhou et al., 2024). High AI awareness was related to career uncertainty and job insecurity as employees cope with the threat of being replaced by AI (Kong et al., 2021). The insecurity also increases stress and emotional exhaustion, leading to counterproductive work behavior (Zhou et al., 2024). It has also been found that high AI awareness can amplify employees’ AI anxiety levels, including learning and job replacement anxieties, as well as sociotechnical blindness (Elfar, 2025). Indeed, these fear-based schemas can subsequently be used to filter information and aid in future appraisal tasks due to their pre-existing nature (Dozois and Beck, 2008; Taylor and Crocker, 1981). This tendency is underpinned by the prominent confirmation bias theory, which posits that people tend to seek, interpret, or distort newly received information to fit and reinforce their pre-existing beliefs (Nickerson, 1998; Wason, 1960). The reinforcement of pre-existing beliefs also implies that attitudes may be resistant to change, particularly when people tend to avoid cognitively effortful restructuring and prefer maintaining the equilibrium of the mind (Cancino-Montecinos et al., 2020).

### 1.3 Perceptual fear in AI counseling

The flipped side of cognitive convenience, however, could be the potentially generalized and biased evaluations of AI performance. Since counseling chatbots share the same nature of AI, people inevitably have similar concerns mentioned earlier about the use of AI in the mental health context. Nevertheless, the use of chatbots in the mental health industry and other sectors cannot be equated. Job replacement would be less likely to occur in the mental health context, given that there is a shortage of resources to meet the demand for mental health services. Counseling chatbots may help address this limitation and complement the roles of human counselors, rather than displacing them (see Table 1).

**Table 1:** Role complementation between counseling chatbots and human counselors.

Features	Counseling chatbots	Human counselors
Accessibility	Chatbots offer accessible emotional support during late-night hours when mood-disturbances are most prevalent (Golder and Macy, 2011).	Human counselors face inherent limitations that preclude ceaseless and round-the-clock services.
Activeness	Both modalities facilitate emotional support by employing active listening techniques, enabling clients to articulate internal experiences (Lee et al., 2017; Prescott et al., 2024).	
Anonymity	Some clients feel more comfortable disclosing thoughts and feelings with a chatbot without the fear of judgement (Aktan et al., 2022; Lucas et al., 2014).	Some clients feel embarrassed disclosing private distressful issues with a human counselor (Aktan et al., 2022; Lucas et al., 2014).
Authenticity	Even with natural language processing and the efficiency of deep learning, chatbots may not be able to offer the authentic emotional connections that humans could provide (Khawaja and Bélisle-Pipon, 2023).	Human counselors are inherently able to offer clients with genuine and authentic emotional connections and rapport (Schnellbacher and Leijssen, 2009).
Flexibility	Chatbots are programmed and trained with data, meaning that they may not be able to handle unexpected situations (Khawaja and Bélisle-Pipon, 2023).	Human counselors have inherently unique intuitions to perceive each client’s subtle cues and emergencies.
Repertoire	Both modalities employ their repository of therapeutic knowledge to formulate responses to clients’ psychological concerns (Chandel et al., 2018).	
Scalability	Chatbots can handle multiple conversations simultaneously without massive financial and human resources.	Traditional human counseling sessions are usually 1:1 (except for group therapies), necessitating massive financial and human resources.
Variety	Chatbots can adaptively deliver diverse therapeutic approaches through analysis of extensive datasets without operational constraints (Bajwa et al., 2021).	The acquisition of proficiency in diverse psychotherapies presents significant challenges within constrained training timelines.

Regarding privacy concerns, chatbots can be developed without relying on existing platforms if developers have sufficient resources or by utilizing strict access controls and internal hosting models. The cybersecurity concerns about AI services may partly stem from cognitive bias or discomfort with unfamiliar systems, as the adoption of AI services can be analogized to traditional counseling or medical consultations, where people typically trust clinical settings to safeguard their sensitive health records despite the inherent risks of data breaches involving third-party cloud storage providers or external vendors. Indeed, research has demonstrated people’s mistrust of computers or algorithms and their preference for information from humans (Dietvorst et al., 2015; Promberger and Baron, 2006). The disparity in perceived trust between conventional and AI-mediated counseling services reflects the potentially biased evaluations people have towards AI.

While algorithmic bias remains a valid concern due to the inherent data-driven nature of AI, the programmatic output can ensure systematic and standardized detection of clients’ needs, reducing inconsistencies that could potentially arise from human judgment. Machine learning algorithms are utilized to deliver contextually relevant responses and employ evidence-based therapeutic techniques grounded in psychological therapies (Balcombe and De Leo, 2022). For example, AI may assist in diagnosing mental illness by identifying relevant patterns in the data (Sun et al., 2023). Given the similar structured nature of AI and Cognitive Behavioral Therapy (CBT), Sebri et al. (2021) found that cognitive psychologists adopting the CBT approach have generally more positive beliefs about the adoption of AI in counseling. Existing empathy-driven chatbots designed with cognitive behavioral principles, such as Woebot and Wysa, have also been suggested to improve users’ mood levels effectively (Fitzpatrick et al., 2017; Inkster et al., 2018). These suggest the benefits of AI algorithms in assisting low-level structured counseling services.

Regarding concerns about chatbots being incapable of reciprocal affect and understanding, which is typically cued by facial expressions and nonverbal body language (Brown and Halpern, 2021), the rapid development of computer-mediated communications has enabled people to communicate online without relying on face-to-face nonverbal cues. As an alternative, emojis, which encompass a wide range of expressions, are created and used to substitute the nonverbal cues in online communication contexts (Boutet et al., 2021; Gesselman et al., 2019; Pfeifer et al., 2022). The rapid advancements in natural language processing (e.g., GPT-4) also allow increasingly sophisticated and more empathetic responses as evaluated by a clinical psychologist (Moell, 2024). In fact, local online counseling platforms like Open Up also offer online textual human counseling services where face-to-face emotions and body language are not involved. Despite the absence of face-to-face nonverbal cues during textual counseling, the platform is recognized by well-known local charities and often appears in queues, reflecting the positive recognition of their mental health support. However, due to service overload, some people cannot access their online services as promptly as intended, and it would be unfortunate to see a prospective client quit reaching out for emotional support.

Given the observed disparity in perceived trust between conventional and AI-mediated counseling services and the lack of nonverbal cues in online textual human counseling services, some questions arise: Does the actual support quality differ between AI and online human textual counseling? Does the perceived support quality of the counseling chatbot differ with different informed labels (i.e., AI vs. human)? Perceptual fear of AI, as coined in this study, refers to the state of having biased and more negative appraisals towards AI performance than its actual capability. Drawing on the confirmation bias theory, different informed labels are expected to elicit varying effects on the perceived support quality of the counseling chatbot. Specifically, people who are told to receive AI support would rate the quality of support more negatively.

### 1.4 Research aims and hypotheses

Before incorporating AI into the mental health industry, it is essential to investigate public attitudes toward AI counseling to predict public acceptance and adoption. While existing research focuses on investigating public attitudes toward AI and the mechanisms of AI anxiety, little is known about the formation of these attitudes and anxieties, particularly the frequency, emotional valence and immersion dimensions of exposure. This study fills this gap by investigating the relationships between prior AI exposures, AI anxiety, general attitudes towards AI, and attitudes towards AI counseling. Despite the growing research on the development and use of AI in the mental health context, the role of bias in shaping support quality evaluations towards counseling chatbots remains unexplored. Therefore, this study also aims to explore the potential existence of perceptual fear influencing people’s support quality ratings. While previous studies exploring public attitudes toward AI, especially in the context of mental health, have been predominantly conducted in Western cultures, this study addresses this gap by focusing on the Asian context, specifically the local Chinese in Hong Kong.

**Figure 1:**
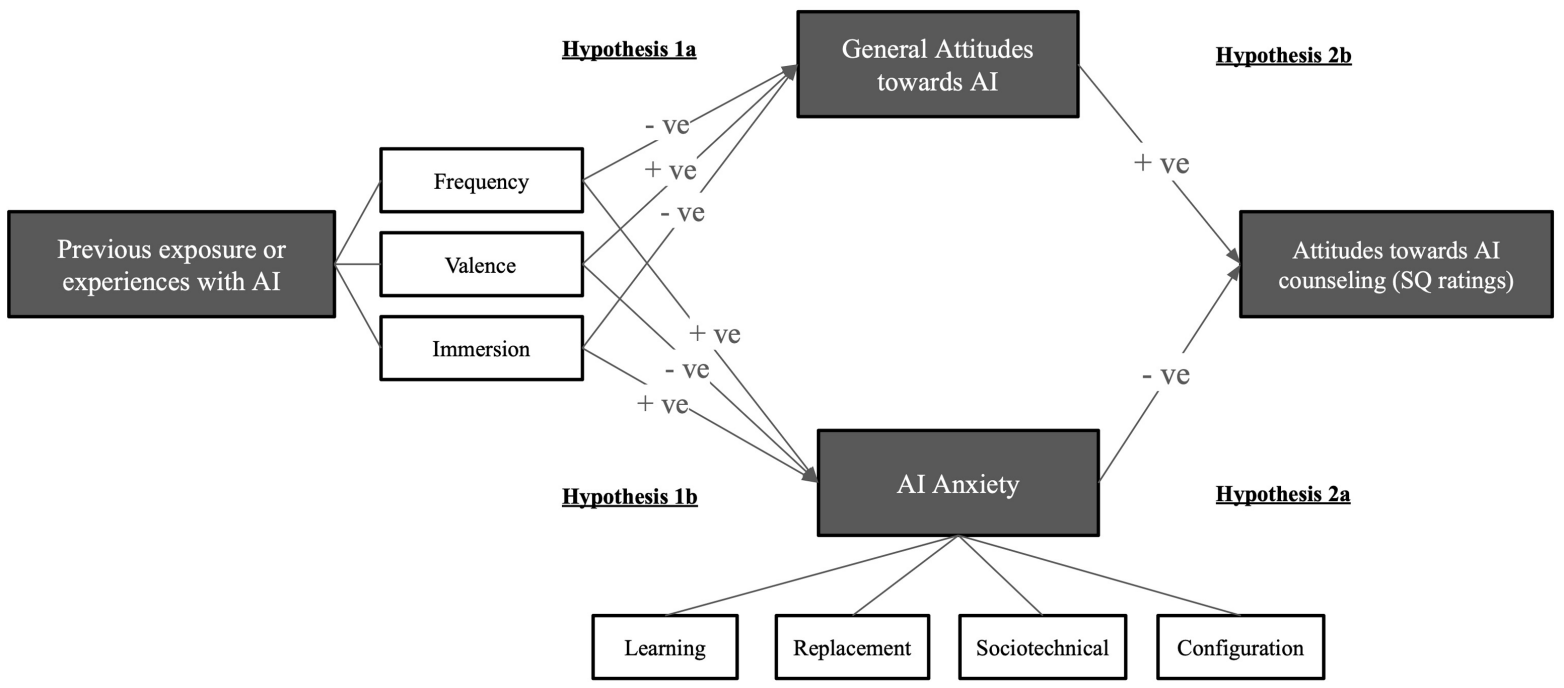
Hypothesized relationships between previous AI exposure, general attitudes towards AI, AI anxiety and SQ ratings.

To mitigate the risk of collecting hypothetical responses from participants without prior experience with chatbot counseling, this study utilized a simulated counseling chatbot to ensure participants had actual engagement with it before providing attitudinal responses. This study also studied the existence of perceptual fear by manipulating perceptual labels “human counseling” or “AI counseling” to investigate whether people’s post-chat support quality ratings, which were also served to indicate their attitudes towards AI counseling, would differ under different informed labels when both groups indeed received support from the same chatbot. Along with the discussions and rationales above, we hypothesized that:

*Hypothesis 1*: The more unpleasant exposure to AI, (a) the more unfavorable general attitudes people have towards AI, and (b) the higher the level of AI anxieties (Figure 1).

*Hypothesis 2*: Due to confirmation bias and maintenance of pre-existing beliefs, (a) the higher the level of AI anxieties, and (b) the more negative general attitudes towards AI, the more negative ratings towards the support quality of counseling chatbot (Figure 1).

*Hypothesis 3*: People would also show no significant change in general attitudes towards AI before and after the chatbot-counseling experience due to confirmation bias and maintenance of pre-existing beliefs.

*Hypothesis 4*: The Told-AI group, who were told to be receiving AI support, would rate support quality more negatively than the Told-Human group’s pre-reveal support quality ratings due to perceptual fear.

*Hypothesis 5*: After the revelation of the true condition, the Told-Human group would show significantly more negative post-reveal support quality ratings than their pre-reveal ratings due to the activation of the salient negative perception of AI.

## 2 Methods

### 2.1 Participants

A total of 161 participants were recruited through social media platforms and the eNotices system of The University of Hong Kong. The inclusion criteria for participants were:

1. Aged 18 or above, due to the ethical considerations of intervention decisions at younger ages (Behnke and Warner, 2002).
2. Native Cantonese speaker, as to facilitate communication effectiveness throughout counseling.
3. Able to read and understand English, since the existing questionnaires employed in this study only have English versions.
4. Have not been diagnosed with any psychiatric or mental disorders.
5. No prior experience in receiving online human or AI counseling, as the experience might influence manipulation effectiveness and perceived support quality of the chatbot.

We welcomed participants of all genders, educational attainments, employment statuses, and job sectors. Participation was voluntary. Participants who had completed all study procedures were awarded HKD50 shopping voucher.

### 2.2 Measures

#### 2.2.1 Sample demographics and characteristics

Information on participants’ gender, age, educational attainment, employment status, and job sector was collected. Participants’ computer expertise (1 = *I can hardly use the computer*, 4 = *I am an expert computer user*), AI knowledge (1 = *0–2 days per week*, 4 = *7 days per week*), and AI usage levels (1 = *I have no knowledge at all*, 4 = I have detailed knowledge) were also collected and rated in four-point Likert scales.

#### 2.2.2 Previous exposures to AI and human counseling

The exposure survey first inquired about the types of AI technologies participants encountered across real-world interactions and simulated exposures (e.g., cinematic portrayals). A total of 20 six-point Likert-scale items were administered, including questions that asked whether participants had been exposed to media (e.g., films, forums, news) that portray AI as undesirable or a villain (1 = *completely disagree*, 6 = *completely agree*). If participants selected an answer other than “completely disagree,” they would rate the frequency of exposure (1 = *rarely*, 6 = *always*), perceived feelings about the experiences (1 = *very unpleasant*, 6 = *very pleasant*), and their perceived immersion in the exposure (1 = *not immersive at all*, 6 = *completely immersive*). It also asked whether they had direct usage experiences with AI products and whether they were aware of some negative information about AI products (1 = *completely disagree*, 6 = *completely agree*). If participants selected an answer other than “completely disagree,” they would rate the frequency of usage and exposure (1 = *rarely*, 6 = *always*) and their feelings about the experiences (1 = *very unpleasant*, 6 = *very pleasant*).

A higher score on frequency items indicated a higher frequency of exposure. A lower score on emotional valence items reflected a higher level of unpleasantness about the experiences. A higher score on the immersion item indicated a higher level of immersion in AI exposure. Questions regarding participants’ exposure to information about human counseling served only to mitigate suspicion concerning the assigned condition of Told-Human group. Thus, the data were not analyzed in this study.

#### 2.2.3 AI anxiety

Considering that nationality and culture may influence AI-related anxiety, the Artificial Intelligence Anxiety Scale (AIAS), which was developed and validated with Chinese populations and possesses good psychometric properties, was utilized in this study to measure participants’ levels of AI anxiety (Wang and Wang, 2022). The scale consists of 21 seven-point Likert-scale items (1 = *not at all*, 7 = *completely*), comprising a four-factor structure— *learning* (items 1–8), *job replacement* (items 9–14), *sociotechnical blindness* (items 15–18), and *AI configuration* (items 19–21). A higher score on each subscale indicates a higher corresponding anxiety level.

#### 2.2.4 General attitudes towards AI

While no existing measures had been developed specifically in the Chinese context by the time of design of this study, the General Attitudes Toward Artificial Intelligence Scale (GAAIS), which was developed by Schepman and Rodway (2020) with good psychometric properties, appeared to be the most widely cited measure for general attitudes towards AI. Consequently, this study utilized it to measure participants’ general attitudes toward AI before and after the support session. The scale contains 21 five-point Likert-scale items (1 = *strongly disagree*, 5 = *strongly agree*), comprising 12 Positive GAAIS items, 8 reversely scored Negative GAAIS items (items 3, 6, 8–10, 16, 20–21), and 1 item for attention check (item 13) to exclude random-clicking responses. A higher score on each subscale indicates a more positive attitude toward AI (Schepman and Rodway, 2020).

#### 2.2.5 Pre-chat survey

Prior to the support session, participants identified one main issue they hoped to work through with the supporter. They also rated their initial perceived stress level regarding the issue with a ten-point semantic differential scale (1 = *not stressful*, 10 = *extremely stressful*). These questions functioned both to prepare participants for their session topic and to measure their initial stress levels for subsequent evaluation of the chatbot’s effectiveness in providing emotional outlets.

#### 2.2.6 Post-chat perceived support quality (SQ) survey

Post-chat SQ ratings were used to reflect participants’ attitudes toward AI counseling. The Told-Human group completed two post-chat SQ surveys (i.e., before and after the revelation of true condition). In the first SQ survey, the group rated their perceived support quality based on the informed label of “human counseling” with 6 ten-point semantic differential scale items measuring (1) perceived relationship quality—“*Do you feel heard, understood and respected?*”; (2) goal—“*Did the counselor work on what you wanted to talk about?*”; (3) approach—“*Is the counselor’s suggested approach a good fit for you?*”; and (4) the overall satisfaction with the session. The 4-item Session Rating Scale (SRS), developed by Duncan et al. (2003) with good psychometric properties, was utilized in this study to evaluate the quality of the therapeutic alliance between the chatbot and users. This short scale was selected because it may help reduce dropout rates and avoid participant fatigue. The items encompass the three core components of the working alliance, as suggested by Bordin (1979), namely emotional bond, shared goals, and consensus on methods or approaches, to promote positive psychological change. Considering the poor mobile compatibility of visual analog scales, particularly due to the difficulty of accurately tapping tiny line marks, this online study replaced the visual analog scale with a ten-point semantic differential scale.

As an additional way to evaluate support quality, we also inquired about participants’ (5) perceived deservingness for another session (1 = *not deserving at all*, 10 = *essentially deserving*). Their post-chat stress level regarding the issue was also measured for subsequent comparison of stress levels. A four-point Likert-scale manipulation check item: “*Were you chatting with a person or an AI?*” (1 = *definitely an AI*, 4 = *definitely a human*) was used to ensure effective manipulation of the condition. Only those selected “*definitely an AI*” were excluded from data analysis.

The second SQ survey for the Told-Human group was identical to that given to the Told-AI group. It included the same five questions assessing support quality and their perceived stress level, but with an informed label of “AI.” As another way to evaluate the effectiveness of the counseling chatbot, we also inquired about their perceived helpfulness of the chatbot (1 = *very harmful*, 7 = *very helpful*). This question was derived from Casey et al. (2013) to predict the perceived helpfulness of e-mental health services.

Participants’ stress levels and perceived helpfulness of the chatbot, which were used to reflect the chatbot’s effectiveness in providing emotional outlets, were analyzed separately from other SQ ratings. A lower post-chat stress level than their initial level reflected the chatbot’s effectiveness in providing emotional outlets. A higher average score on other SQ ratings indicated a more positive perceived support quality of the counseling chatbot.

### 2.3 AI counseling chatbot

A simulated online counseling chatbot was developed using the Azure OpenAI GPT-4 model (1106-preview version). It is a deep learning model designed for natural language processing, leveraging trained data to generate human-like textual responses. The coding of the chatbot is openly available at https://github.com/socathie/my-peer.

#### 2.3.1 System characteristics

**Figure 2:**
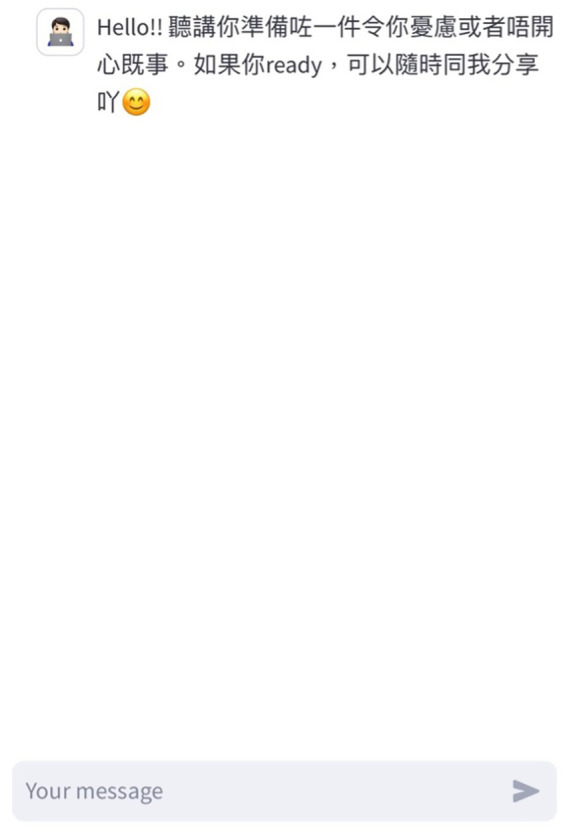
The same distraction-free chat interface was used by all participants during the experiment.

Consistent chatbot configuration settings were employed for all participants. They were exposed to the same system characteristics (Table 2) and a distraction-free user interface (Figure 2). Since reading a message in a language different from our mother tongue could easily result in misinterpretation of messages (Buarqoub, 2019), the chatbot’s output language was colloquial Cantonese to reduce language barriers and enhance communication effectiveness throughout the session. To enhance the deception effectiveness of the Told-Human group, the chatbot’s response time was calibrated according to the average reading and typing speed of Chinese users. According to Wang et al. (2019), the reading speed of native Chinese is 259 characters/min when reading Chinese characters, while the Chinese typing speed of native Chinese is 57.1 characters/min (Chen and Gong, 1984; Fong and Minett, 2012).

**Table 2:** System characteristics of simulated counseling chatbot.

Characteristics	System settings
Language of output	Colloquial Cantonese^a^
Speed	Reading: 259 characters/min
	Typing: 57 characters/min
Maximum Tokens per response	800
Temperature^b^	0.7
Top P^c^	0.95
Stop sequence^d^	“?” and “? “
Frequency penalty	None
Presence penalty	None

a The chatbot intermittently generates English responses contingent upon users’ use of lexical terms in their inputs.

b Temperature: A lower temperature yields more repetitive and deterministic responses.

c Top P: A lower Top P narrows the model’s token selection to likelier tokens.

d Stop sequence determines how the chatbot’s response ends in a desired way.

#### 2.3.2 Turn-taking conversations and chat monitoring

**Figure 3:**
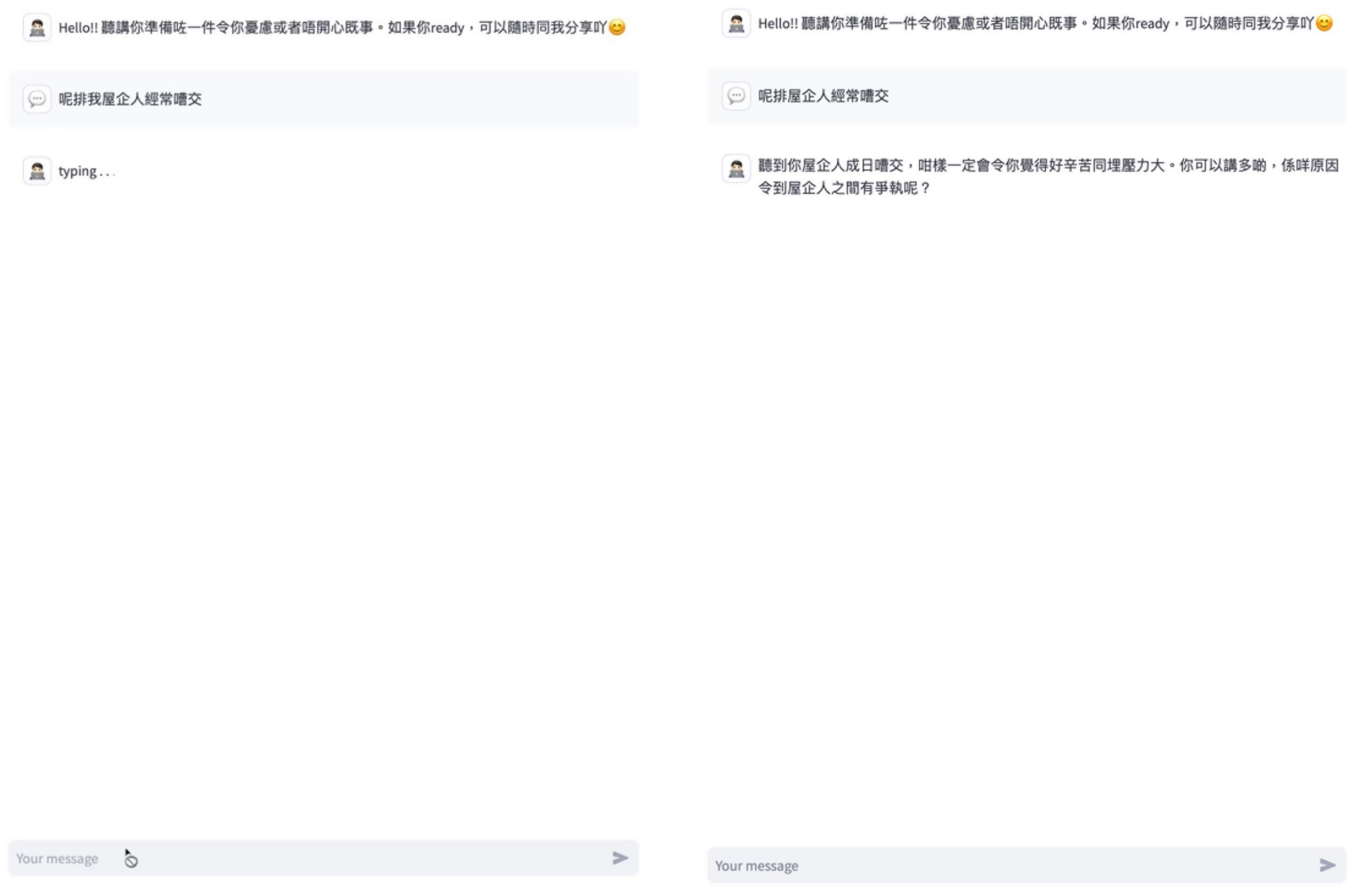
The system displayed a real-time typing signal and temporarily disabled the input field while participants awaited a response.

Like any other contemporary language models, the interaction followed a turn-taking structure (i.e., participant message followed by an AI response). During the AI response generation period, the interface presented a real-time typing signal, with the participant’s input field temporarily deactivated. Only when they received an AI response could they type their next message or response (Figure 3). To prevent platform misuse and maintain active participant presence during the experiment, the researcher monitored real-time chat records using PromptLayer with the exact time of each turn shown in the system.

#### 2.3.3 Chatbot system message

All participants interacted with the same standardized chatbot system messages (i.e., backend AI instructions) (Appendix A). The instructions were tested through trials using Azure OpenAI Studio, with the aim of generating more natural and emotionally supportive responses. The simulated chatbot designed for this study did not involve sophisticated or comprehensive instructions to AI since this study prioritizes investigations of perceptual label effects on perceived support quality of the chatbot, rather than its therapeutic effectiveness in addressing participants’ psychological issues.

Some CBT techniques were utilized as the primary counseling approach during the session. CBT is well-studied to be one of the most systematic and evidence-based approaches for counseling (David et al., 2018; Hofmann et al., 2012), as well as the most popular technique for handling stress-related experiences (Cuijpers et al., 2021; David et al., 2018; Etzelmueller et al., 2020). CBT is also particularly suitable for time-constrained studies given that its structured nature minimizes dynamic variability.

### 2.4 Procedure

All surveys used in this study were created and distributed online using Qualtrics. Potential participants clicked on the invitation link, where they filled in the consent form and eligibility test to register for the study. Since deception was necessary to investigate the impact of perceptual labels on participants’ attitudes toward AI counseling, participants were initially informed that the study investigates public attitudes toward human textual counseling and AI counseling, so they would have the chance to receive support from a human counselor. The researcher contacted eligible participants online to confirm their acknowledgment of the study procedure and the conditions they were assigned for this study. The randomization was done by coin flipping. Participants were assigned to the Told-Human group if the coin landed on heads, and to the alternative condition if it landed on tails. Meanwhile, they were assigned unique participant IDs for privacy and progress tracking purposes.

All participants were then given a link to the first set of questionnaires, where they filled in the exposure survey, AIAS, GAAIS, and the pre-chat survey. Since the content of the first questionnaire was mostly related to AI, the Told-Human group was informed that those questions were included to examine whether previous AI exposure would affect their attitudes towards human textual counseling and further confirm their assigned condition. Afterwards, participants were scheduled for the chat session according to their availability and were reminded that (1) the chat conversation would be recorded and kept strictly confidential, (2) the session would be conducted in Cantonese, accepting only a little English for participants who are used to talk in mixed language, (3) they could use any device they found comfortable, but were remined to maintain continuous interface engagement to prevent automatic session recommencement. To avoid suspicion, the researcher informed the Told-Human group that the session’s recommencement indicates a possibility of reconnection with a new counselor on duty. All participants were also informed that (4) they would receive a reminder after 50 min through a message or a call from the researcher.

The reminder message was sent again an hour before the scheduled session, during which the researcher also reminded participants about the issue they had previously stated as the session’s focus. If participants wished to work on a different issue before the session, they were asked to indicate the new issue and their corresponding stress levels before the chat. These amendments were updated accordingly by the researcher. Upon entering the chat platform, participants were presented with a message written in colloquial Chinese: “*Hello!! I heard that you have prepared something that makes you worried or sad. If you are ready, feel free to share it with me* 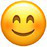.” During the 50-min chat, the researcher recorded the time and monitored the chat starting from when a turn-taking message from the participant and AI was issued until the end of the session. If no turn-taking interaction was observed for over 10 min, the researcher would show concerns about their status. Upon completing the 50-min session, the researcher sent a message or initiated an alarming call and reminded participants not to use the platform beyond the experimental period. This signaled the closure of the platform on their end.

**Figure 4:**
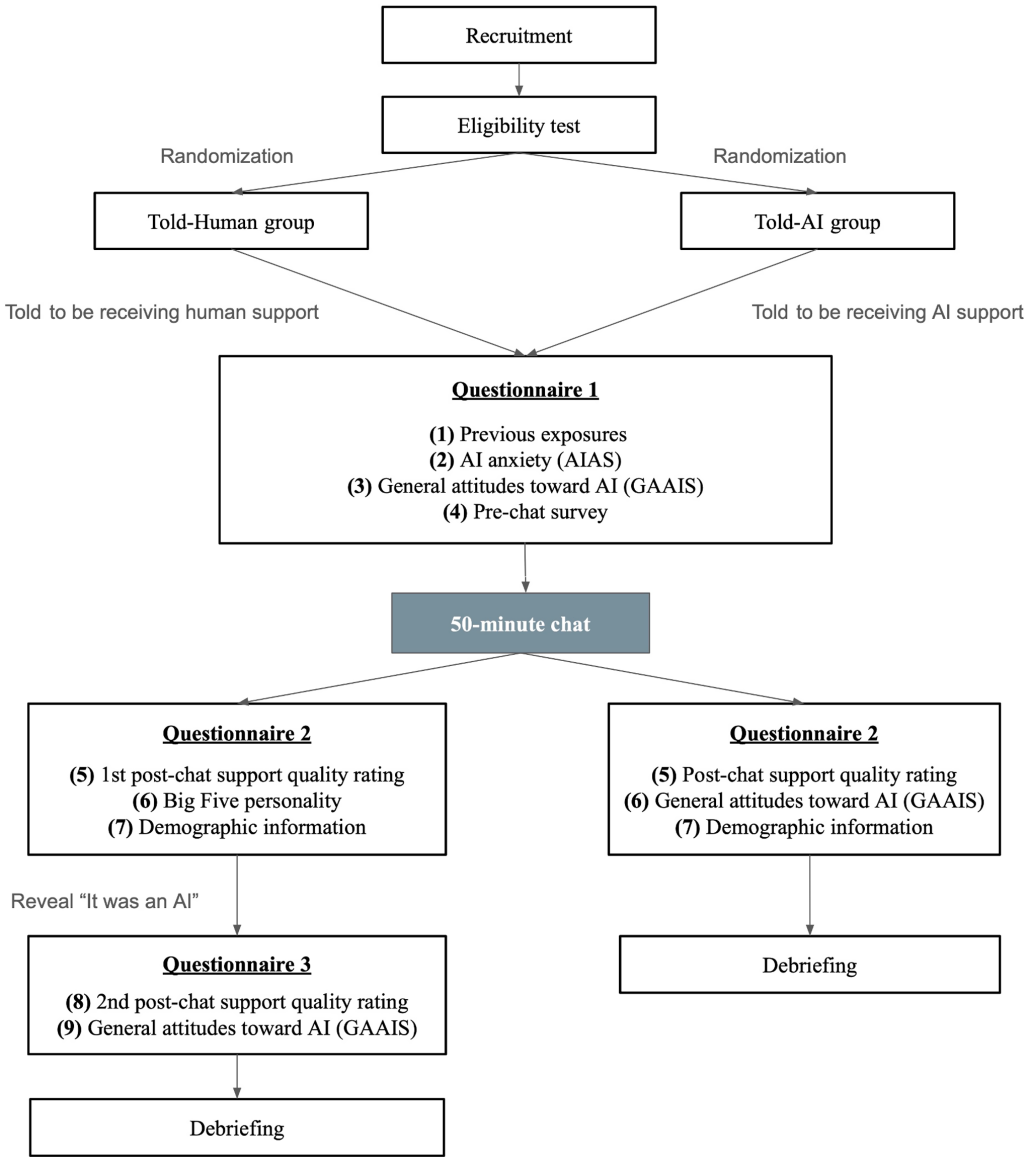
Flowchart illustrating the study procedure.

Immediately after the chat, the Told-Human group was asked to complete the second set of questionnaires within 12 h. It included the first SQ survey, BFI-10 and demographic survey. Upon completion, they were revealed that the “person” they chatted with online was AI all the time and were asked to complete the third set of questionnaires within 12 h after the revelation. It included the second SQ survey and the second GAAIS. For the Told-AI group, they were asked to complete a post-chat survey within 12 h after the chat, which included the SQ survey, the second GAAIS and the demographic survey. Eventually, both groups were debriefed via an online debriefing form, which disclosed the study’s true objectives. Upon completing all procedures (Figure 4), participants were welcome to contact the researchers for any queries regarding the study and were scheduled to collect the shopping voucher in person.

### 2.5 Statistical analyses

SPSS 29.0 was used for conducting all statistical analyses and descriptive outputs. Correlations for the relationships of interest were computed using Pearson’s correlation analysis. Independent sample t-tests were used to compare between-group SQ ratings and the perceived helpfulness of the chatbot. Paired sample t-tests were used to examine general attitude change before and after the chat with AI, the within-group change of SQ ratings in the Told-Human group, as well as the changes in stress levels in both groups. All data were bootstrapped with 5,000 samples to obtain 95% confidence intervals (CI) and are bias-corrected and accelerated (BCa). Results were considered significant if the upper and lower values of the 95% confidence interval did not contain 0 between them and met the significance level of 5% (*p* < 0.05). All statistical outputs were rounded to two decimal places, except for bootstrapped CI bounds, which were reported to three decimal places to maintain precision in interpreting significance. Since Rammstedt and John’s (2007) BFI-10 for the Told-Human group was included for extended purposes only, the data were not analyzed in this study.

## 3 Results

### 3.1 Sample demographics and descriptive statistics

**Figure 5:**
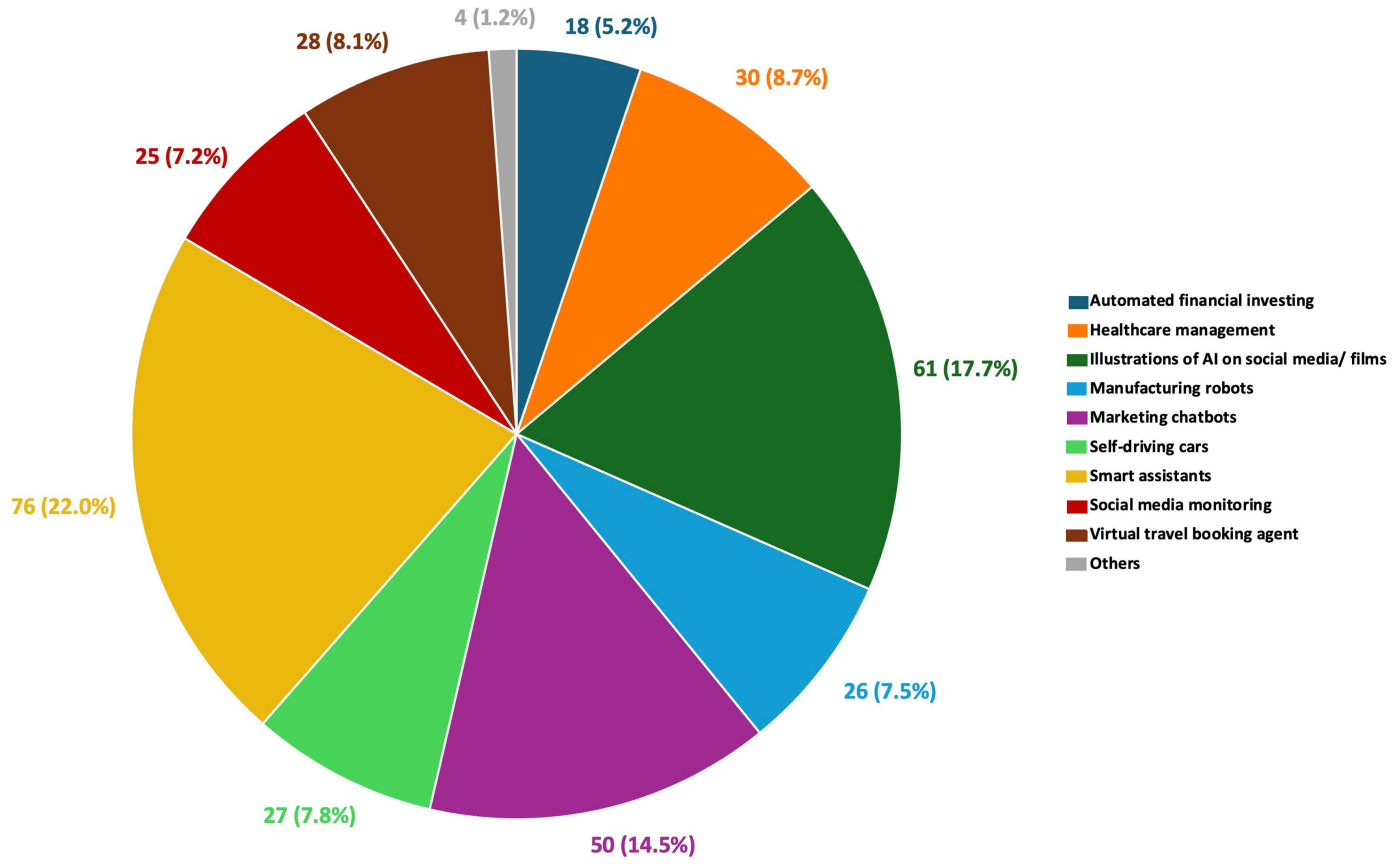
A pie chart showing the distribution of AI technologies encountered by participants.

Fifty-one participants failed to pass the attention check (*n* = 2), manipulation check (*n* = 2), dropped out (*n* = 20), or did not chat for at least 40 min (*n* = 27). After excluding their data, valid responses from 110 participants (*n* = 55 per group) were included in the data analysis for this study. In the Told-Human group, 18 (32.7%) are male and 3 (5.5%) prefer not to say. Ages ranged from 18 to 71 (*M* = 29.93, SD = 11.38). In the Told-AI group, 16 (29.1%) are male, and 3 (5.5%) prefer not to say. Their ages ranged from 18 to 60 (*M* = 28.13, SD = 9.16). The gender proportion observed in both groups aligns with the typical finding that women are more likely to seek psychological help than men (Nam et al., 2010). Note that since the participants’ demographic data were collected after the experiment, and all other excluded data can no longer be retrieved since only valid responses were retained for data analysis, the demographics of the analyzed sample could not be compared with those of the original sample to inform the full representativeness of the final dataset. Nonetheless, there were no significant differences in demographic characteristics between the Told-Human and Told-AI groups (Table 3). Figure 5 presents the types of AI technologies participants encountered.

**Table 3:** Sample demographics and characteristics.

Characteristics *N* (%)		Told-Human (*n* = 55)	Told-AI (*n* = 55)
Educational level	Primary	0	0
	Secondary	3 (5.5%)	4 (7.3%)
	Diploma/ Associate	3 (5.5%)	4 (7.3%)
	Bachelor	39 (70.9%)	37 (67.3%)
	Master	10 (18.2%)	8 (14.5%)
	Doctorate	0	2 (3.6%)
Job sector	Accountancy, banking and finance	7 (12.7%)	3 (5.5%)
	Arts	1 (1.8%)	1 (1.8%)
	Business and marketing	5 (9.1%)	4 (7.3%)
	Education	14 (25.5%)	12 (21.8%)
	Engineering	3 (5.5%)	3 (5.5%)
	Healthcare and hospitality	12 (21.8%)	16 (29.1%)
	Information technology	1 (1.8%)	2 (3.6%)
	Law	2 (3.6%)	1 (1.8%)
	Law enforcement	3 (5.5%)	1 (1.8%)
	Others	7 (12.7%)	12 (21.8%)
Job status	Full-time	32 (58.2%)	33 (60%)
	Part-time	8 (14.5%)	3 (5.5%)
	Retired	2 (3.6%)	2 (3.6%)
	Self-employed	1 (1.8%)	3 (5.5%)
	Student	12 (21.8%)	13 (23.6%)
	Unemployed	0	1 (1.8%)
Computer expertise level	Hardly use the computer	0	0
	Slightly below-average computer user	5 (9.1%)	4 (7.3%)
	Average computer user	47 (85.5%)	40 (72.7%)
	Expert computer user	3 (5.5%)	11 (20%)
AI usage level	0–2 day (s) per week	36 (65.5%)	31 (56.4%)
	3–4 days per week	14 (25.5%)	14 (25.5%)
	5–6 days per week	3 (5.5%)	6 (10.9%)
	7 days per week	2 (3.6%)	4 (7.3%)
AI knowledge level	No knowledge at all	2 (3.6%)	7 (12.7%)
	Little knowledge	27 (49.1%)	23 (41.8%)
	Some knowledge	26 (47.3%)	23 (41.8%)
	Detailed knowledge	0	2 (3.6%)

Participants seldom or occasionally had exposure to AI (*M* = 2.97, SD = 0.80) and reported neutral emotional valence (*M* = 3.52, *SD* = 0.80) and moderate immersion in their exposures (*M* = 3.20, SD = 1.12). They reported relatively low levels of AI learning (*M* = 2.38, SD = 0.89), replacement (*M* = 3.25, SD = 0.98), sociotechnical (*M* = 3.17, SD = 0.91) and AI configuration anxieties (*M* = 2.51, SD = 1.08). At baseline, they reported neutral attitudes towards AI, as indicated by both positive (*M* = 3.48, SD = 0.52) and negative (*M* = 2.99, SD = 0.50) scales. To ensure that the informed condition labels did not affect participants’ reports of previous exposure, ratings of AI anxiety and their attitudes towards AI in a confounding manner, independent t-tests were conducted, and the results showed no observed differences between the groups (Table 4).

**Table 4:** No observed effect of informed condition on between-group pre-chat ratings.

Variables	Mean difference	*t* (108)	*p*	BCa 95% CI		Effect Size (Hedges’ g)
				Lower	Upper	
Frequency	0.16	1.08	0.29	−0.132	0.455	0.80
Valence	0.16	1.04	0.30	−0.133	0.454	0.80
Immersion	0.22	1.02	0.31	−0.210	0.645	1.13
Learning anxiety	−0.01	−0.07	0.95	−0.336	0.307	0.90
Replacement anxiety	0.20	1.07	0.29	−0.152	0.562	0.98
Sociotechnical anxiety	0.29	1.66	0.10	−0.042	0.627	0.91
Configuration anxiety	−0.02	−0.12	0.91	−0.410	0.365	1.09
Pre-chat attitudes (positive-scale)	−0.03	−0.32	0.75	−0.231	0.161	0.52
Pre-chat attitudes (negative-scale)	−0.03	−0.28	0.78	−0.212	0.159	0.51

BCa, Bias-corrected and accelerated.

### 3.2 Reliability and validity of measures

In terms of reliability, given the short subscales in the exposure scale, Cronbach’s alpha was not computed. The mean inter-item correlations were computed on the frequency (*r* = 0.33) and emotional valence subscales (*r* = 0.37) to assess reliability and validity. Results indicated adequate internal consistency and convergent validity (Briggs and Cheek, 1986; Clark and Watson, 1995).

Cronbach’s *α* was calculated for existing scales used in this study to assess internal consistency. The replacement of the visual analog scale with a ten-point semantic differential scale for the four original items in SRS resulted in Cronbach’s α of 0.94. It demonstrated improved consistency compared to the original SRS using analog scale (0.88). The Cronbach’s α for the SQ survey after adding the deservingness item was 0.92. This high degree of internal consistency reflects that the five items correlate highly with one another, much like the SRS (Duncan et al., 2003). The Cronbach’s α for learning anxiety (*α* = 0.93), replacement (*α* = 0.89), sociotechnical blindness (*α* = 0.84), and AI configuration (*α* = 0.90) yielded similar favorable reliability results to those originally reported for the AIAS (Wang and Wang, 2022). Likewise, the Cronbach’s α for positive-scale attitudes (*α* = 0.88) and negative-scale attitudes (*α* = 0.78) in our study yielded similar results to those in the original study (Schepman and Rodway, 2020).

In terms of validity, since immersion was assessed with a single item due to its context-specific nature in media exposure, while it precludes consistency analysis, we computed its correlation with emotional valence as an alternative validation strategy. Results showed that a stronger immersion is associated with stronger emotional responses, *r* = 0.41, *p* < 0.001, BCa 95% CI = [0.176, 0.599]. For SQ ratings, the mean inter-item correlation of 0.71 reflected favorable evidence for convergent validity in the 5-item SQ.

The computed mean inter-item correlations for learning anxiety (*r* = 0.58), replacement (*r* = 0.57), sociotechnical blindness (*r* = 0.57), and AI configuration (*r* = 0.75) showed minor deviations from the original study but yielded good validity results (Wang and Wang, 2022). However, when assessing validity for GAAIS in our study, our results of Exploratory Factor Analysis yielded a 4-factor solution accounting for 47.71% variance, diverging from Schepman and Rodway’s (2020) 2-factor structure (41.6%). While their study cleanly separated positive (12 items) and negative (8 items) attitudes, our analysis revealed that nine positive items were in Factor 1, five negative items in Factor 2, and that Factors 3 and 4 had cross-loading items. The factor correlation between Factor 1 and Factor 2 was 0.16, which was weaker than the original (*r* = 0.59). Despite this, the mean inter-item correlation showed that positive-scale items (*r* = 0.36) and negative-scale items (*r* = 0.30) demonstrated acceptable validity of the subscales.

### 3.3 Bivariate correlation analyses

Participants’ unpleasant AI exposure, as indicated by frequency, emotional valence and immersion, were correlated with their attitudes towards AI, validating hypothesis 1a. Significant negative relationships were observed between (1) frequency and negative-scale attitudes, and between (2) immersion and negative-scale attitudes, as well as post-chat positive-scale attitudes. A significant positive relationship was shown between valence and positive-scale attitudes (Table 5). Hypothesis 1b was validated. Specifically, significant positive relationships were found between (1) frequency of exposure and sociotechnical blindness, and between (2) immersion and configuration anxiety, as well as a significant negative relationship between emotional valence and learning anxiety (Table 5).

**Table 5:** Bivariate correlation between previous exposure, general attitudes towards AI and AI anxiety.

Variables		*r*	*p*	Bootstrap		
				*SE*	BCa 95% CI	
					Lower	Upper
Frequency	Pre-Chat Attitudes (Positive)	0.17	0.08	0.08	0.013	0.332
	Post-Chat Attitudes (Positive)	0.01	0.92	0.09	−0.173	0.180
	Pre-Chat Attitudes (Negative)	−0.28**	0.003	0.11	−0.481	−0.079
	Post-Chat Attitudes (Negative)	−0.20*	0.04	0.11	−0.390	−0.010
	Learning Anxiety	0.05	0.63	0.08	−0.121	0.207
	Replacement Anxiety	0.09	0.36	0.09	−0.089	0.272
	Sociotechnical Blindness	0.24*	0.01	0.08	0.061	0.402
	Configuration Anxiety	0.04	0.65	0.09	−0.142	0.232
Valence	Pre-Chat Attitudes (Positive)	0.35**	<0.001	0.08	0.180	0.520
	Post-Chat Attitudes (Positive)	0.21*	0.03	0.08	0.044	0.381
	Pre-Chat Attitudes (Negative)	−0.05	0.58	0.12	−0.295	0.200
	Post-Chat Attitudes (Negative)	−0.10	0.28	0.10	−0.284	0.083
	Learning Anxiety	−0.24*	0.01	0.09	−0.400	−0.076
	Replacement Anxiety	−0.15	0.12	0.08	−0.311	0.004
	Sociotechnical Blindness	−0.03	0.79	0.08	−0.197	0.135
	Configuration Anxiety	−0.17	0.08	0.09	−0.341	0.002
Immersion	Pre-Chat Attitudes (Positive)	−0.10	0.29	0.10	−0.280	0.081
	Post-Chat Attitudes (Positive)	−0.24*	0.01	0.09	−0.395	−0.071
	Pre-Chat Attitudes (Negative)	−0.35**	<0.001	0.10	−0.526	−0.137
	Post-Chat Attitudes (Negative)	−0.33**	<0.001	0.09	−0.480	−0.153
	Learning Anxiety	0.15	0.12	0.10	−0.039	0.329
	Replacement Anxiety	0.19	0.05	0.09	0.006	0.361
	Sociotechnical Blindness	0.10	0.29	0.09	−0.058	0.267
	Configuration Anxiety	0.37**	<0.001	0.08	0.206	0.516

Positive, positive-scale; Negative, negative-scale (reverse-scored). BCa, Bias-corrected and accelerated. *p < 0.05. **p < 0.01.

For exploratory purposes, participants were classified as Gen Z (*n* = 62) if their age was between 18 and 27, and as non-Gen Z for ages above 27. This study found potential generational differences when separate Pearson correlations revealed a significant negative relationship between emotion valence and learning anxiety in Gen Z, *r* = −0.27, *p* = 0.04, BCa 95% CI = [−0.486, −0.020], but not in non-Gen Z, *r* = −0.22, *p* = 0.14, BCa 95% CI = [−0.471, 0.073]. The negative relationship between immersion and post-chat positive-scale attitudes was also specific to Gen Z, *r* = −0.29, *p* = 0.02, BCa 95% CI = [−0.501, −0.041], but not in non-Gen Z, *r* = −0.16, *p* = 0.27, BCa 95% CI = [−0.426, 0.129]. Only non-Gen Z showed a significant positive relationship between frequency of exposure and sociotechnical blindness, *r* = 0.40, *p* = 0.005, BCa 95% CI = [0.127, 0.612], but not in Gen Z, *r* = 0.05, *p* = 0.68, BCa 95% CI = [−0.199, 0.300].

Hypothesis 2a was refuted. SQ ratings were not related to learning anxiety, *r* = −0.16, *p* = 0.10, BCa 95% CI = [−0.312, −0.004], replacement anxiety, *r* = −0.08, *p* = 0.42, BCa 95% CI = [−0.277, 0.119], sociotechnical blindness, *r* = 0.04, *p* = 0.72, BCa 95% CI = [−0.180, 0.235], and configuration anxiety, *r* = −0.13, *p* = 0.18, BCa 95% CI = [−0.295, 0.030]. Additional analysis on the relationship between AI anxiety and general attitudes towards AI showed notable negative correlations between all types of AI anxieties and attitude subscales, suggesting that when individuals’ AI anxiety levels were related to their general attitudes towards AI, it was not necessarily related to their ratings towards AI counseling.

Hypothesis 2b was refuted as not all general attitudes towards AI were significantly related to SQ ratings. Specifically, SQ ratings were not related to pre-chat positive-scale attitudes, *r* = 0.08, *p* = 0.44, BCa 95% CI = [−0.107, 0.259], pre-chat negative-scale attitudes, *r* = 0.05, *p* = 0.58, BCa 95% CI = [−0.114, 0.223], and post-chat negative-scale attitudes, *r* = 0.12, *p* = 0.22, BCa 95% CI = [−0.072, 0.297]. Only the post-chat attitudes in the positive scale showed a notable positive relationship with SQ ratings, *r* = 0.43, *p* < 0.001, BCa 95% CI = [0.250, 0.589].

### 3.4 Change in general attitudes towards AI

Hypothesis 3 was validated. For both Told-AI and Told-Human groups, their attitudes towards AI did not differ before and after the chat. For the Told-Human group, no difference was shown in their pre-chat (*M* = 3.46, SD = 0.54) and post-chat attitudes (*M* = 3.58, *SD* = 0.43) in the positive scale, *t* (54) = −1.88, *p* = 0.07, BCa 95% CI = [−0.245, −0.003], with Hedges’ correction of 0.49, as well as their pre-chat (*M* = 2.97, SD = 0.55) and post-chat attitudes (*M* = 2.99, SD = 0.55) in the negative scale, *t* (54) = −0.29, *p* = 0.77, BCa 95% CI = [−0.136, 0.098], with Hedges’ correction of 0.47. Likewise, for the Told-AI group, no difference was shown in their pre-chat (*M* = 3.50, SD = 0.49) and post-chat attitudes (*M* = 3.48, SD = 0.68) in the positive scale, *t* (54) = 0.22, *p* = 0.83, BCa 95% CI = [−0.127, 0.162] with Hedges’ correction of 0.56, as well as their pre-chat (*M* = 3.00, SD = 0.45) and post-chat attitudes (*M* = 3.09, SD = 0.54) in the negative scale, *t* (54) = −1.34, *p* = 0.19, BCa 95% CI = [−0.207, 0.039], with Hedges’ correction of 0.48.

### 3.5 Effect of perceptual labels on between-group SQ ratings

**Figure 6:**
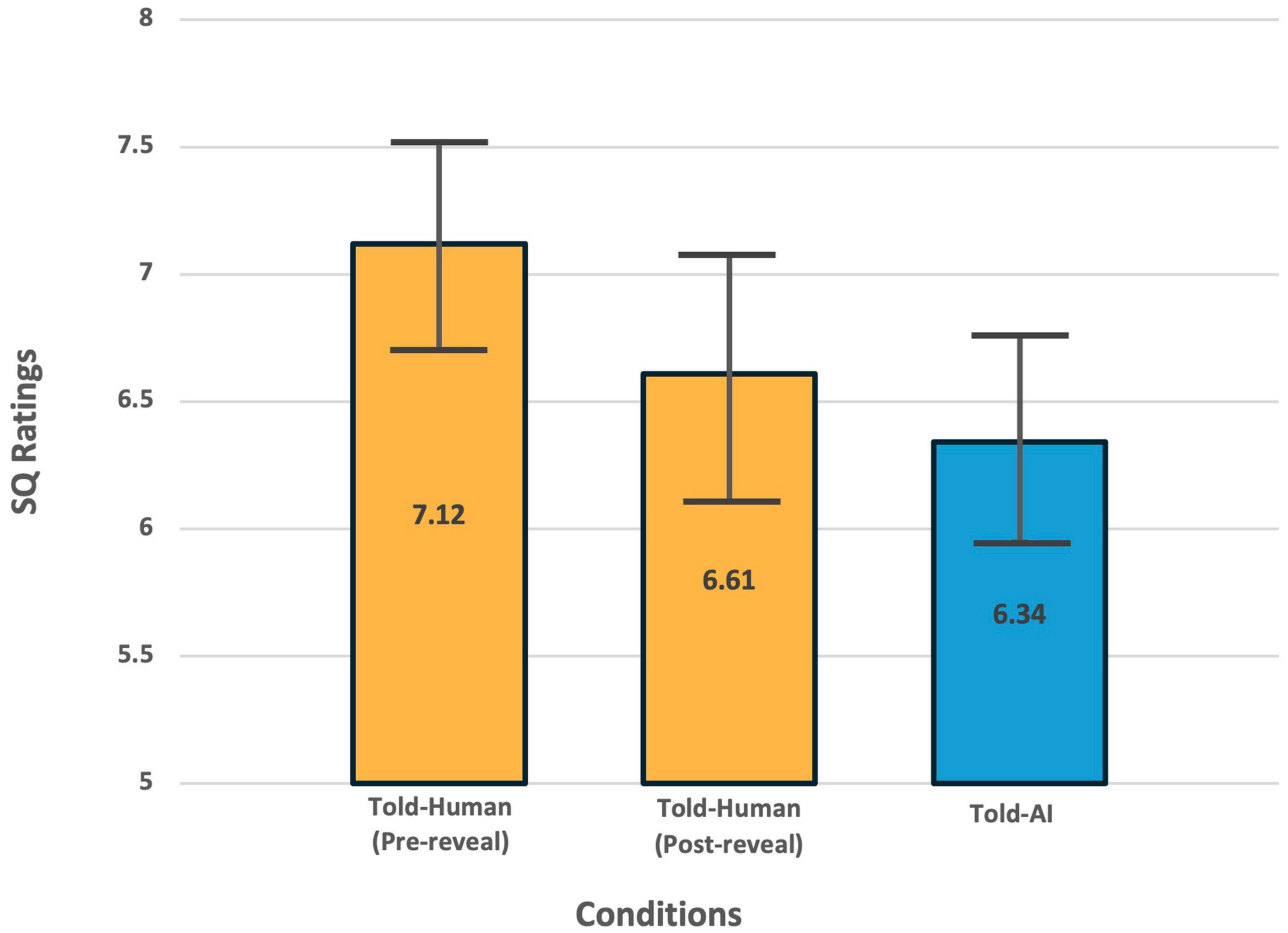
Significant between-group (Told-Human pre-reveal vs Told-AI) and within-group (Told-Human pre-reveal vs post-reveal) differences in perceived counseling chatbot support quality, despite all participants interacted with the same chatbot. Support quality ratings included participants’ evaluations of perceived relationship, goal achievement, fit of approach, and overall satisfaction with the session.

Hypothesis 4 was validated. The Told-AI group’s post-chat SQ ratings (*M* = 6.34, *SD* = 1.56) were significantly less favorable than the post-chat pre-reveal SQ ratings of the Told-Human group (*M* = 7.12, SD = 1.53), *t* (108) = 2.64, *p* = 0.009, BCa 95% CI = [0.186, 1.342], with Hedges’ correction of 1.55. This suggested that individuals’ perceived support quality of counseling chatbot notably differed due to different perceptions (i.e., human or AI) activated and that the Told-AI group rated support quality more negatively due to their perceptual fear of AI (Figure 6). Descriptive statistics on each component of support qualities are shown in Table 6.

**Table 6:** Descriptive statistics of AI counseling support qualities.

Conditions	Variables	*M*	SD
Told-Human (Pre-reveal)	Relationship	7.53	1.70
	Goal	7.64	1.68
	Approach	6.93	1.86
	Overall Satisfaction	7.22	1.61
	Deservingness	6.29	2.18
Told-Human (Post-reveal)	Relationship	7.00	2.06
	Goal	6.89	2.02
	Approach	6.64	1.99
	Overall Satisfaction	6.76	1.94
	Deservingness	5.76	2.36
Told-AI	Relationship	6.55	1.80
	Goal	6.58	1.78
	Approach	6.24	1.89
	Overall Satisfaction	6.45	1.53
	Deservingness	5.89	2.10

### 3.6 Within-group change of SQ ratings in the Told-Human group

Results validated hypothesis 5 when the Told-Human group rated significantly more negatively after being told that they were receiving support from an AI (*M* = 6.61, SD = 1.84) than when they thought they were receiving support from a human (*M* = 7.12, SD = 1.53), *t* (54) = 4.08, *p* < 0.001, BCa 95% CI = [0.302, 0.756], with Hedges’ correction of 0.94, further supporting the hypothesis that the perceptual difference affects people’s perceived support quality of the counseling chatbot. Additional findings have revealed that the Told-Human group’s post-reveal SQ ratings (*M* = 6.61, SD = 1.84) did not differ from the Told-AI group’s SQ ratings (*M* = 6.34, *SD* = 1.56), *t* (108) = 0.83, *p* = 0.41, BCa 95% CI = [−0.383, 0.858], with Hedges’ correction of 1.71 (Figure 6).

### 3.7 Stress levels and chatbot’s helpfulness

**Figure 7:**
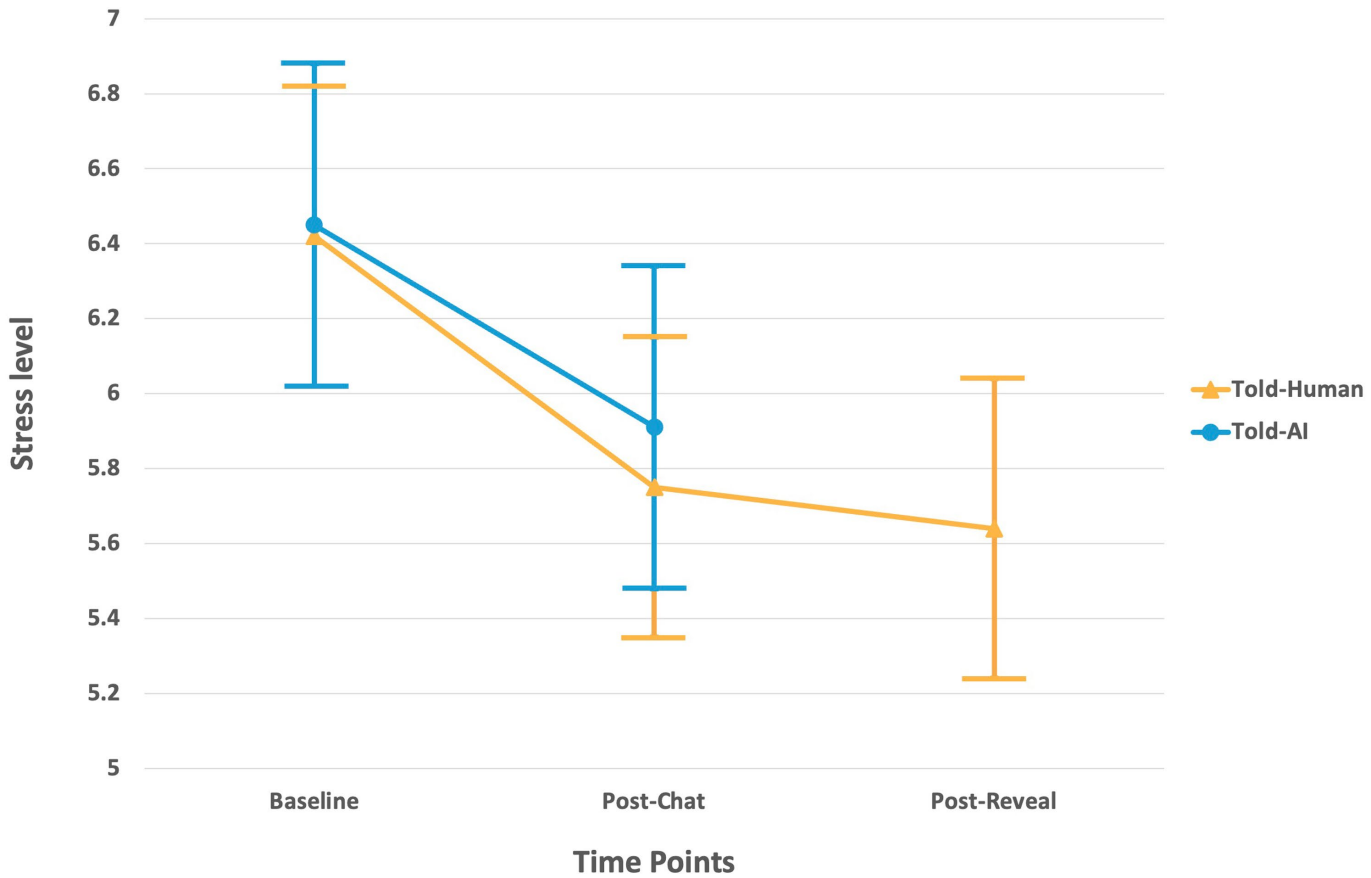
A significant reduction in stress levels was observed in both groups after they received support from the counseling chatbot. The Told-Human group showed no significant difference in stress levels between pre- and post-reveal time points.

Participants generally reported a medium-high initial stress level regarding their concerned issue (*M* = 6.44, SD = 1.60). As an alternative to evaluating the effectiveness of the counseling chatbot, participants’ initial stress levels regarding their issues were compared to their post-chat stress levels (Figure 7). In the Told-AI group, results showed a significant reduction in post-chat stress levels (*M* = 5.91, SD = 1.83) when compared to initial stress level (*M* = 6.45, SD = 1.60), *t* (54) = 2.31, *p* = 0.03, BCa 95% CI = [0.073, 1], with Hedges’ correction of 1.78, reflecting the effectiveness of the counseling chatbot in providing emotional outlets even when people knew that they were receiving AI support.

The Told-Human group also showed a significant reduction in stress levels after the chat when they thought they were chatting with a human (*M* = 5.75, *SD* = 1.62) than initial stress levels (*M* = 6.42, SD = 1.62), *t* (54) = 2.27, *p* = 0.03, BCa 95% CI = [0.109, 1.236], with Hedges’ correction of 2.23. Their post-reveal stress levels (*M* = 5.64, SD = 1.48) had no significant difference from their pre-reveal stress levels, *t* (54) = 0.70, *p* = 0.49, BCa 95% CI = [−0.164, 0.436], with Hedges’ correction of 1.17, reflecting that even when the group realized that they were receiving support from an AI, the revelation of the true condition (or the activation of AI perception) did not affect their stress-level ratings as the other SQ ratings did.

Participants regarded the helpfulness of the counseling chatbot as neutral. The Told-Human group (*M* = 4.65, SD = 1.27) and the Told-AI group (*M* = 4.40, SD = 1.03) did not differ in perceiving the chatbot’s helpfulness, *t* (108) = 1.16, *p* = 0.25, BCa 95% CI = [−0.161, 0.660], with Hedges’ correction of 1.16.

## 4 Discussion

Although the knowledge and development of AI in Hong Kong are quite robust, the United States has a larger scale, more resources, and a faster pace of innovation for AI development (Maslej et al., 2025). The difference in resource availability, investment, and industry presence may limit locals’ exposure to the latest discussions and challenges surrounding AI. These explain the limited exposure to AI, neutral emotional valence, and moderate immersion in our sample. Given the limited perceived personal and social relevance, the sample had neutral attitudes towards AI and relatively low levels of AI anxieties.

Despite adequate validity, the minor deviations in the validity results of AIAS from the original study suggested the need for further examination of item-level performance. Regarding the validity deviations from the original studies of GAAIS, the 4-factor solution of the GAAIS found in this study suggests that the original structure may not fully replicate in our sample. The deviation might be attributable to the cultural differences in how attitudes towards AI manifest in Asians compared to Schepman and Rodway’s (2020) UK sample. Future cross-cultural validation studies may inform whether the GAAIS’s factor structure holds across cultures.

### 4.1 Emotionally charged statements and negativity salience

Consistent with the justifications using Pavlovian conditioning, fight-or-flight reactions to threats (Ekman, 2009), and the findings about message internalization (Green and Brock, 2000; Valkenburg and Peter, 2013), the significant relationships between previous exposure and attitudes towards AI support the expectation that a higher frequency, more negative emotional valence and stronger immersion of previous exposures are related to the development of more negative attitudes towards AI. From a theoretical standpoint, this study aligns with related studies that demonstrated the influence of prior exposure on attitudes towards AI (Hasan et al., 2021; Kirkpatrick et al., 2023; Kirkpatrick et al., 2024). This study distinguishes itself by thoroughly investigating the dimensions of exposure. Specifically, a higher frequency of unpleasant exposures to AI and higher immersion during the experiences are related to more agreement with negative-scale items (e.g., the unethical use of AI, the erroneous nature of AI, and its dangerous nature). A more negative emotional valence of exposure is also related to less agreement towards the positive-scale items (e.g., “*AI systems can perform better than humans*,” “*AI can provide new economic opportunities*”).

Given that frequency, emotional valence, and immersion are all carried by emotions, the one-to-one relationships between previous exposure and a particular subscale of attitudes reflect the emotional attraction between individuals’ experiences and attitudes and also align with previous findings about negativity salience (Robertson et al., 2023; Zollo et al., 2015). For example, the relationship between negative emotional valence and disagreement towards positive-scale items (rather than agreement towards negative-scale items) could be explained by negativity salience and our inherent emotional tendency to oppose or strike back at something we find “unright.” If the sample had some unpleasantly charged feelings in AI exposure, they could be emotionally triggered by the positive statements about AI and strike back by rating more negatively towards these pleasantly charged statements, while also reassuring their pre-existing beliefs about AI (Nickerson, 1998; Wason, 1960). It results in a more salient positive relationship between emotional valence and positive-scale attitudes. Since frequency and immersion also carry emotionally charged mechanisms (i.e., conditioned feelings about AI and the emotional bonding during immersive experiences), the salience of negativity and emotional attraction may explain their negative relationships with negative-scale attitudes.

While the overall sample showed that a stronger immersion was related to significantly more unfavorable post-chat rather than pre-chat positive-scale attitudes, exploratory analyses revealed this effect was significant only in Gen Z. A person with low immersion in unpleasant experiences is less affected or restricted by pre-existing impressions about AI when receiving the chatbot’s support since their attitude formations are less influenced by the impact of immersion. In other words, they are more likely to unfreeze their attitudes towards AI after receiving chatbot counseling, resulting in more apparent attitude changes after the session. This is especially true for Gen Z. Given their fewer life experiences and exposure to the world than non-Gen Z, they generally have more flexible opinions and attitudes. It aligns with and is supported by previous studies on the effects of age on neuroplasticity (Hedden and Gabrieli, 2004), as well as the greater adaptability and openness to change observed in Gen Z or younger individuals (Fuchs et al., 2024; Visser and Krosnick, 1998). In contrast, stronger immersion is associated with more rigid maintenance of pre-existing attitudes due to confirmation bias (Nickerson, 1998; Wason, 1960), resulting in steady or unnoticeable changes in attitudes after receiving the chatbot’s support. Thus, immersion has a stronger and more salient negative relationship with post-chat rather than pre-chat positive-scale attitudes.

### 4.2 Urging for cautious AI developments

To the researchers’ knowledge, related studies investigating the relationship between exposure and AI anxiety have been conducted in organizational contexts (Elfar, 2025; Kong et al., 2021; Zhou et al., 2024). This study addresses the gap by focusing on the general public and examining exposure dimensions to enrich broader discourse on the formation of AI anxieties. Consistent with related studies about AI awareness and AI anxiety, individuals’ frequency, emotional valence, and immersion in exposure are each related to particular AI anxieties. Specifically, a higher frequency of AI exposure is associated with greater sociotechnical blindness, which is particularly pronounced among non-Gen Z individuals. Aligning with cognitive dissonance theory (Festinger, 1957) and related studies on ambivalence and uncertainty (Buttlar et al., 2024; van Harreveld et al., 2009), individuals with greater exposure to dual-perspective information about AI may experience uncertainty regarding the societal implications of AI-driven change. Indeed, the insecurity of change has been a central concept in social psychology, suggesting that humans are inherently conservative and prioritize tradition over societal change (Jost, 2015). It is attributable to the desire to sustain the comfortable state secured by collective interests, shared reality, and a sense of belonging, as well as the maintenance of mental equilibrium (Cancino-Montecinos et al., 2020). Given that non-Gen Z individuals were not raised in a fully digitalized society like Gen Z, frequent exposure to dual-perspective information about AI could intensify this conservative human nature and make them more insecure about AI’s advancement over time.

Nevertheless, reporting unpleasant incidents related to AI is necessary, as it prompts the need for regulations and remedial strategies to address the issues presented. To minimize the negative impact of sociotechnical anxiety on people’s attitudes and adoption behaviors, AI developers should take the lead in emphasizing that humans are always the masters of technology and social change. While developers are enthusiastic about the progressive advancements in AI, focusing on iterative improvements to existing systems, rather than rapid and radical deployment, may reduce failure rates and their subsequent reporting. These considerations become especially vital when accounting for the negative relationship between emotional valence and learning anxiety found in this study, which aligns with Elfar’s (2025) findings that high AI awareness could amplify employees’ AI learning anxiety. The development of insecurity could adversely affect people’s decisions or confidence in learning AI, subsequently hindering AI literacy. The relationship was particularly noticeable to Gen Z since they are the generation more likely to learn AI to keep pace with societal advancements, unlike non-Gen Z individuals who may already have established careers. It therefore underscores the need for gradual development and prioritizes improvements on existing programs while allowing sufficient time for societal adaptation.

Immersion in AI exposure is found to correlate positively with the development of AI configuration anxiety. Consistent with the findings about message internalization (Green and Brock, 2000; Valkenburg and Peter, 2013), people who have more emotional investments in exposures that illustrate AI as undesirable (e.g., movies that depict AI as a self-conscious destructive villain, or news about job replacement by AI) are more likely to fear humanoid AI configuration and concern about its increasingly sophisticated development in performing human abilities. Since configuration anxiety could signal the formation of negative attitudes towards AI, we advocate for the responsible development of AI through industry regulations and guidelines to alleviate public fears and concerns about AI’s humanoid features.

### 4.3 Necessitating the development of AI counseling attitude scale

As mentioned, SQ ratings towards the counseling chatbot only serve as indicators or partial reflections of attitudes towards AI counseling. Given the absence of relationships between most general attitudes and AI counseling SQ ratings in our study, developing an attitudinal scale for AI counseling is essential to inform more sound investigations of the relationship and provide more complete reflection of attitudes towards AI counseling. Several subscales that measure support quality, counseling accessibility, ethical considerations, user experience, and clients’ autonomy when engaging with the chatbot would be examples to help predict public attitudes and the adoption of AI counseling in a more comprehensive manner. The development of such a scale could also enable practitioners in related fields to systematically evaluate AI counseling and identify areas for improvement, thereby better meeting clients’ needs. It could also inform the development of guidelines to ensure the proper use of AI in counseling and allow researchers to conduct comparative studies on attitudes in various populations, perspectives (e.g., clients vs. counselors), and cultures, to gain a better understanding of the societal implications of AI counseling.

Likewise, the nonsignificant relationships between AI anxieties and SQ ratings suggest that the domains or conceptual frameworks underpinning perceived support satisfaction towards counseling chatbots may not be adequate to reflect complete attitudes towards AI counseling. Another possible reason is that since the AIAS and GAAIS were designed and considered AI in an all-in-one manner, they did not fully cover the features or conceptual frameworks of using AI in the counseling context. Reviewing back to the relationship between general attitudes and SQ ratings, a notable positive correlation is observed only between post-chat positive-scale attitudes and SQ ratings. The only significant result in the “post-chat” ratings is that the support quality was assessed only after receiving the chatbot’s support. The “positive-scale” items are more related to psychological satisfaction, which directly shares a similar conceptual nature with well-being and support satisfaction in AI counseling (e.g., Q4 “*Artificially intelligent systems can help people feel happier*” and Q11 “*Artificial Intelligence can have positive impacts on people’s well-being*”). Given the conceptual relevance, post-chat positive-scale attitudes are related to AI counseling SQ ratings.

In light of this, a more comprehensive examination of the relationship between AI anxieties and SQ ratings could be conducted by enriching the prospective AI counseling attitude scale with conceptual frameworks related to AI anxiety (e.g., potential concerns over role displacement in counseling, discomfort with communication and interaction with AI). The incorporation of these complementary items could potentially yield more fruitful study results regarding their relationship.

### 4.4 Emergence of perceptual fear in AI counseling

Although support quality did not provide a complete picture of people’s attitudes towards AI counseling, it provided information about individuals’ perceived support satisfaction regarding the working alliance between chatbots and humans. To the researchers’ knowledge, there has been no prior attempt to investigate perceptual fear or biased support quality appraisals towards counseling chatbots. Our study aligns with previous related studies about people’s mistrust of information from computers and algorithms (Dietvorst et al., 2015; Promberger and Baron, 2006). Our between-group results of SQ ratings demonstrated that people generally have biased appraisals towards AI in the counseling context. The perceived support satisfaction of the Told-Human and Told-AI groups significantly differs even when both groups indeed received the same chatbot support.

In accordance with the confirmation bias theory, the nonsignificant within-group difference between the pre-and-post-chat general attitudes and the less favorable support quality ratings towards the “AI” label reflect the human propensity to maintain mental equilibrium (Cancino-Montecinos et al., 2020), and people tend to interpret or distort newly received information to reinforce pre-existing beliefs (Nickerson, 1998; Wason, 1960). In other words, the Told-AI group exhibited biased and more negative appraisals towards AI performance than its actual capability. Perceptual fear was also observed when the Told-Human group rated support quality more negatively after being revealed the true condition. It implies that even when they knew that the support was the same regardless of the revelation, the effect of perceptual fear on perceived experiences was salient.

While the emergence of perceptual fear introduces challenges in obtaining unbiased support quality ratings within the AI counseling context, concealing AI support is unacceptable as clients have the right to know the kind of support they would receive and “who” they would share the issues with. Future studies may investigate the impact of perceptual fear on perceived support satisfaction in countries that deploy AI counseling support tools (e.g., the United States), so as to inform potential strategies for mitigating perceptual fear in the context of AI counseling.

### 4.5 Implicit effectiveness and explicit reservations about helpfulness

While people generally showed more biased and negative evaluations of AI, the significant reduction in stress levels is consistent with previous findings on the effectiveness of counseling chatbots in improving users’ mood (Fitzpatrick et al., 2017; Inkster et al., 2018), which reflects AI’s capability to provide emotional outlets. However, people had reservations about its helpfulness, which may be due to the blockage by their pre-existing beliefs.

The presentation difference of questions regarding stress levels and helpfulness projects the contradiction between implicit effectiveness and explicit reservations about helpfulness. Perceived stress levels were assessed before (i.e., initial stress level) and after the chat (i.e., post-chat stress levels), meaning that each stress level rating was separated by some time. Participants were unlikely to recall their initial stress levels, nor did they have a “standard” in mind to project their pre-existing beliefs about AI. In other words, they rated post-chat stress levels based on their true experience after the chat (and the revelation). In contrast, the one-time item about perceived helpfulness offered a more straightforward way to project pre-existing beliefs because of its explicit presentation. After all, stress levels would be more reliable indicators of the counseling chatbot’s effectiveness in providing emotional outlets, given that its presentation is less contaminated by perceptual fear.

### 4.6 Limitations and other future directions

Despite the adequate reliability and validity reflected by mean inter-item correlations, the exposure subscales (i.e., frequency and emotional valence) have not been properly validated. Findings about exposure should be interpreted as preliminary and replicated with validated measures in future work. As mentioned earlier, the demographics of the analyzed sample could not be compared with those of the original sample, as the demographic data were not collected until the last set of questionnaires, and only valid responses were retained for data analyses. Future work should collect demographic data at baseline and retain it to allow for the assessment of sample representativeness.

Since this study only used perceived support qualities as indicators of attitudes toward AI counseling, it may not sufficiently provide a complete picture of public attitudes toward AI counseling. Furthermore, given that the data were collected from local Chinese in Hong Kong, the results may only reflect the local context. Future studies could consider conducting cross-cultural examinations of perceptual fear in AI counseling and developing a comprehensive attitudinal scale to measure public attitudes toward AI counseling.

Considering the typical working hours (i.e., 8–10 h) for full-time workers in Hong Kong, the time limit for participants to complete each survey was set at 12 h after the survey link was issued to provide sufficient time for completion while minimizing the dropout rate. The 12-h latency, however, may reduce accuracy stemming from short-term memory effects. Meanwhile, since this study focuses on chatbot counseling, where prospective clients typically interact with it in real-life settings, experimenting in a laboratory may not yield more representative results. To facilitate memory retention, future studies could impose a stricter time limit for ratings; however, caution should be exercised to minimize drop-out rates. They could also recruit a larger sample size to strengthen the generalizability of results.

The system message given to the counseling chatbot in this study generally employed the CBT approach due to its well-researched effectiveness and its structured nature that is compatible with AI. Future explorations could utilize AI to deliver counseling techniques that involve more dynamics and variations (e.g., psychodynamic and humanistic approaches) to examine whether similar findings can be obtained. Further explorations are encouraged to utilize different support quality measures to examine the existence of perceptual fear in AI counseling, as well as to conduct sentiment and thematic analysis of the conversations to compare the emotional tone and choice of words used in the Told-Human and Told-AI groups, to inform about their relative willingness or openness to disclose with a “human” or “AI.”

## 5 Conclusion

Consistent with previous theories and studies, individuals’ previous unpleasant exposures to AI were associated with the development of AI anxieties and negative attitudes towards AI. The development of AI anxieties was not related to individuals’ perceived support quality of the counseling chatbot due to potential differences in conceptual frameworks. Only the post-chat attitudes on the positive scale were related to the perceived support quality of the chatbot, given their similar nature in terms of emotional well-being.

Aligning with the confirmation bias theory, no significant change in general attitudes towards AI was observed in either group. The observed existence of perceptual fear of AI adversely affected people’s perceived support quality of the counseling chatbot. Nevertheless, the significant reduction in stress levels has demonstrated the capability of counseling chatbots in providing emotional support. This study highlights the importance of accounting for the influence of individuals’ pre-existing beliefs on the perceived support quality of counseling chatbots. Future cross-cultural studies with a larger sample may shed more light by investigating dynamic intervention approaches and conducting sentiment and thematic analyses of client-chatbot conversations.

## Data Availability

The raw data supporting the conclusions of this article will be made available by the authors, without undue reservation.
